# (1*S*)-1,2-*O*-Benzyl­idene-α-d-glucurono-6,3-lactone

**DOI:** 10.1107/S1600536809002876

**Published:** 2009-01-31

**Authors:** Sarah F. Jenkinson, Daniel Best, Alexander C. Weymouth-Wilson, Robert A. Clarkson, George W. J. Fleet, David J. Watkin

**Affiliations:** a Department of Organic Chemistry, Chemistry Research Laboratory, Department of Chemistry, University of Oxford, Oxford OX1 3TA, England; b Dextra Laboratories Ltd, Science and Technology Centre, Whiteknights Road, Reading RG6 6BZ, England; c Department of Chemical Crystallography, Chemistry Research Laboratory, Department of Chemistry, University of Oxford, Oxford OX1 3TA, England

## Abstract

X-ray crystallographic analysis has established that the major product from the protection of d-glucoronolactone with benzaldehyde is (1*S*)-1,2-*O*-benzyl­idene-α-d-glucurono-6,3-lactone, C_13_H_12_O_6_, rather than the *R* epimer. The crystal structure exists as O—H⋯O hydrogen-bonded chains of mol­ecules lying parallel to the *a* axis. The absolute configuration was determined by the use of d-glucuronolactone as the starting material.

## Related literature

For related literature on the synthesis of protected d-glucuronolactone, see: Sheldrick *et al.* (1983 ▶); Macher *et al.* (1979 ▶); Shah (1969 ▶). For literature related to the use of acetonide-protected d-glucuronolactone as an inter­mediate in the synthesis of (*a*) other sugars, see: Bleriot *et al.* (1997 ▶); Dax *et al.* (1991 ▶); Ke *et al.* (2003 ▶); Masaguer *et al.* (1997 ▶); (*b*) imino sugars, see: Dax *et al.* (1990 ▶); (*c*) sugar amino acids, see: Bashyal *et al.* (1986 ▶, 1987 ▶); (*d*) many other bioactive targets, see: Kitahara *et al.* (1974 ▶); Austin *et al.* (1987 ▶); Witty *et al.* (1990 ▶); Shing & Tsui (1992 ▶); Yoda *et al.* (2002 ▶). For the original NMR studies on benzylidene-protected glucorono­lactone, see Csuk *et al.* (1984 ▶).
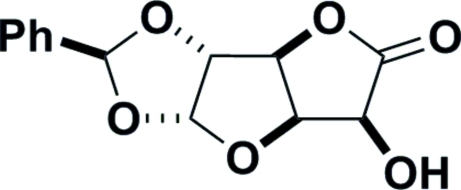


## Experimental

### 

#### Crystal data

C_13_H_12_O_6_
*M* *_r_* = 264.23
Monoclinic, 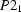

*a* = 5.6329 (1) Å
*b* = 7.8943 (2) Å
*c* = 13.3182 (3) Å
β = 99.9545 (9)°
*V* = 583.32 (2) Å^3^
*Z* = 2
Mo *K*α radiation
μ = 0.12 mm^−1^
*T* = 150 K
0.60 × 0.50 × 0.30 mm

#### Data collection

Nonius KappaCCD area-detector diffractometer
Absorption correction: multi-scan (*DENZO*/*SCALEPACK*; Otwinowski & Minor, 1997 ▶) *T*_min_ = 0.88, *T*_max_ = 0.96
8275 measured reflections
1418 independent reflections
1341 reflections with *I* > 2σ(*I*)
*R*_int_ = 0.022

#### Refinement

*R*[*F* ^2^ > 2σ(*F* ^2^)] = 0.027
*wR*(*F* ^2^) = 0.068
*S* = 0.96
1418 reflections
172 parameters
1 restraint
H-atom parameters constrained
Δρ_max_ = 0.20 e Å^−3^
Δρ_min_ = −0.18 e Å^−3^

### 

Data collection: *COLLECT* (Nonius, 2001 ▶); cell refinement: *DENZO*/*SCALEPACK* (Otwinowski & Minor, 1997 ▶); data reduction: *DENZO*/*SCALEPACK*; program(s) used to solve structure: *SIR92* (Altomare *et al.*, 1994 ▶); program(s) used to refine structure: *CRYSTALS* (Betteridge *et al.*, 2003 ▶); molecular graphics: *CAMERON* (Watkin *et al.*, 1996 ▶); software used to prepare material for publication: *CRYSTALS*.

## Supplementary Material

Crystal structure: contains datablocks global, I. DOI: 10.1107/S1600536809002876/lh2760sup1.cif

Structure factors: contains datablocks I. DOI: 10.1107/S1600536809002876/lh2760Isup2.hkl

Additional supplementary materials:  crystallographic information; 3D view; checkCIF report

## Figures and Tables

**Table 1:** Hydrogen-bond geometry (Å, °)

*D*—H⋯*A*	*D*—H	H⋯*A*	*D*⋯*A*	*D*—H⋯*A*
O7—H71⋯O1^i^	0.86	1.97	2.811 (3)	165

Symmetry code: (i) 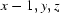.

**Fig. 1.**
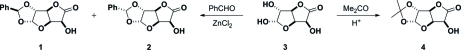
Synthetic scheme

**Fig. 2.**
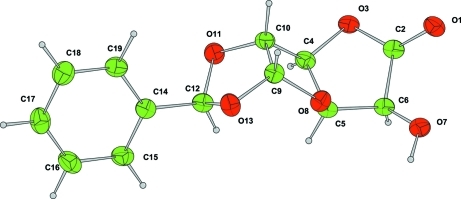
The title compound with displacement ellipsoids drawn at the 50% probability level. H atoms are shown as spheres of arbitary radius.

**Fig. 3.**
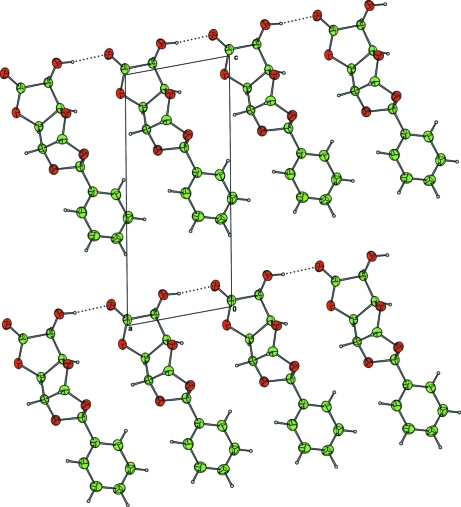
Packing diagram of the title compound projected along the b-axis. Hydrogen bonding is indicated by dotted lines.

**Fig. 4.**
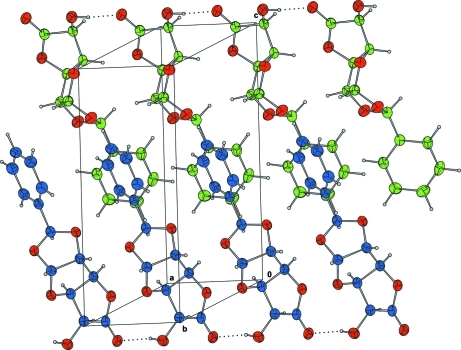
Packing diagram showing alternating layers of hydrogen bonded chains of molecules.

## supplementary crystallographic information

### Comment

*D*-Glucuronolactone 3 (Fig. 1), the only cheaply availably uronic acid, reacts with acetone in the presence of an acid catalyst to form the acetonide 4 (Sheldrick *et al.*, 1983). With only a single unprotected hydroxyl group, the lactone 4 provides convenient access to C-5 of *D*-glucose and has long been used as a versatile intermediate for the synthesis of other sugars (Bleriot *et al.*, 1997; Dax *et al.*, 1991; Ke *et al.*, 2003; Masaguer *et al.*, 1997), imino sugars (Dax *et al.*, 1990), sugar amino acids (Bashyal *et al.*, 1986, 1987) and many other bioactive targets (Kitahara *et al.*, 1974; Austin *et al.*, 1987; Witty *et al.*, 1990; Shing & Tsui, 1992; Yoda *et al.*, 2002). Reaction of 3 with benzaldehyde in the presence of zinc chloride gives a high yield of the benzylidene protected lactones in which the epimers are formed in a ratio of approximately 5:1 (Macher *et al.*, 1979; Shah, 1969). The configuration of the benzylidene acetal has previously been investigated by NMR experiments which suggest that 1, which is the major product, has the 1,2(*S*)-configuration (Csuk *et al.*, 1984). The crystallographic analysis confirms that this assignment is correct and that the major product is 1. Although as yet there have been no examples of the use of the benzylidene acetals 1 and 2 as synthetic intermediates, it is likely there will be cases where the use of a benzylidene group, which can be removed by hydrogenation, will have a significant advantage over the acetonide 4, where strong acid must be used to remove the protecting group.

The title compound (Fig. 2) exists as alternating layers of hydrogen bonded chains of molecules lying parallel to the *a*-axis (Fig. 3, Fig. 4). Only classical hydrogen bonding has been considered. The absolute configuration was determined by the use of *D*-glucuronolactone as the starting material.

### Experimental

The title compound was recrystallized by vapour diffusion from a mixture of ethyl acetate and cyclohexane: m.p. 419.5–421.5 K; [α]_D_^20^ +67 (*c*, 1.0 in acetone) (Macher *et al.*, 1979).

### Refinement

In the absence of significant anomalous scattering, Friedel pairs were merged and the absolute configuration was assigned from the starting material.

The H atoms were all located in a difference map, but those attached to C atoms were repositioned geometrically. The H atoms were initially refined with soft restraints on the bond lengths and angles to regularize their geometry (C—H in the range 0.93–0.98, O—H = 0.82 Å) and *U*_iso_(H) (in the range 1.2–1.5 times *U*_eq_ of the parent atom), after which the positions were refined with riding constraints.

### Crystal data

**Table:**

C_13_H_12_O_6_	*F*(000) = 276
*M**_r_* = 264.23	*D*_x_ = 1.504 Mg m^−^^3^
Monoclinic, *P*2_1_	Mo *K*α radiation, λ = 0.71073 Å
Hall symbol: P 2yb	Cell parameters from 1368 reflections
*a* = 5.6329 (1) Å	θ = 5–27°
*b* = 7.8943 (2) Å	µ = 0.12 mm^−^^1^
*c* = 13.3182 (3) Å	*T* = 150 K
β = 99.9545 (9)°	Plate, colourless
*V* = 583.32 (2) Å^3^	0.60 × 0.50 × 0.30 mm
*Z* = 2	

### Data collection

**Table:**

Nonius KappaCCD area-detector diffractometer	1341 reflections with *I* > 2σ(*I*)
graphite	*R*_int_ = 0.022
ω scans	θ_max_ = 27.5°, θ_min_ = 5.2°
Absorption correction: multi-scan (*DENZO*/*SCALEPACK*; Otwinowski & Minor, 1997)	*h* = −7→7
*T*_min_ = 0.88, *T*_max_ = 0.96	*k* = −10→10
8275 measured reflections	*l* = −17→17
1418 independent reflections	

### Refinement

**Table:**

Refinement on *F*^2^	Primary atom site location: structure-invariant direct methods
Least-squares matrix: full	Hydrogen site location: inferred from neighbouring sites
*R*[*F*^2^ > 2σ(*F*^2^)] = 0.027	H-atom parameters constrained
*wR*(*F*^2^) = 0.068	Method = modified Sheldrick *w* = 1/[σ^2^(*F*^2^) + (0.04*P*)^2^ + 0.13*P*], where *P* = [max(*F*_o_^2^,0) + 2*F*_c_^2^]/3
*S* = 0.96	(Δ/σ)_max_ = 0.009
1418 reflections	Δρ_max_ = 0.20 e Å^−^^3^
172 parameters	Δρ_min_ = −0.18 e Å^−^^3^
1 restraint	

### Fractional atomic coordinates and isotropic or equivalent isotropic displacement parameters (Å^2^)

**Table:**

	*x*	*y*	*z*	*U*_iso_*/*U*_eq_	
O1	1.1555 (2)	0.28908 (18)	1.09521 (9)	0.0317	
C2	1.0013 (3)	0.2487 (3)	1.02461 (12)	0.0257	
O3	1.04981 (19)	0.2312 (2)	0.92986 (9)	0.0296	
C4	0.8321 (3)	0.1868 (2)	0.85789 (12)	0.0263	
C5	0.6246 (3)	0.2272 (2)	0.91452 (12)	0.0246	
C6	0.7382 (3)	0.2089 (2)	1.02587 (12)	0.0259	
O7	0.6540 (2)	0.3147 (2)	1.09703 (9)	0.0334	
O8	0.5720 (2)	0.40215 (18)	0.89088 (9)	0.0285	
C9	0.6089 (3)	0.4347 (2)	0.79011 (12)	0.0265	
C10	0.8011 (3)	0.3081 (2)	0.76761 (12)	0.0267	
O11	0.6942 (2)	0.2266 (2)	0.67619 (9)	0.0323	
C12	0.4413 (3)	0.2413 (2)	0.66815 (12)	0.0269	
O13	0.4052 (2)	0.40025 (19)	0.71549 (9)	0.0307	
C14	0.3210 (3)	0.2382 (2)	0.55862 (12)	0.0266	
C15	0.1004 (3)	0.1573 (3)	0.53152 (14)	0.0320	
C16	−0.0149 (3)	0.1563 (3)	0.43042 (15)	0.0379	
C17	0.0921 (3)	0.2338 (3)	0.35636 (14)	0.0374	
C18	0.3143 (3)	0.3125 (3)	0.38315 (14)	0.0368	
C19	0.4288 (3)	0.3166 (3)	0.48437 (14)	0.0321	
H41	0.8338	0.0667	0.8363	0.0325*	
H51	0.4805	0.1554	0.8912	0.0314*	
H61	0.7293	0.0846	1.0439	0.0312*	
H91	0.6542	0.5570	0.7843	0.0323*	
H101	0.9551	0.3623	0.7612	0.0324*	
H121	0.3793	0.1500	0.7071	0.0326*	
H151	0.0235	0.1040	0.5830	0.0404*	
H161	−0.1724	0.1016	0.4108	0.0448*	
H171	0.0114	0.2344	0.2852	0.0453*	
H181	0.3876	0.3631	0.3302	0.0450*	
H191	0.5853	0.3725	0.5058	0.0382*	
H71	0.5030	0.2905	1.0901	0.0522*	

### Atomic displacement parameters (Å^2^)

**Table:**

	*U*^11^	*U*^22^	*U*^33^	*U*^12^	*U*^13^	*U*^23^
O1	0.0237 (6)	0.0355 (8)	0.0344 (6)	0.0018 (5)	0.0009 (5)	−0.0001 (5)
C2	0.0234 (7)	0.0216 (7)	0.0319 (8)	0.0030 (7)	0.0044 (6)	0.0031 (7)
O3	0.0193 (5)	0.0384 (7)	0.0316 (6)	0.0026 (5)	0.0051 (4)	0.0001 (6)
C4	0.0213 (7)	0.0265 (8)	0.0306 (8)	0.0011 (6)	0.0036 (6)	−0.0021 (7)
C5	0.0209 (7)	0.0227 (8)	0.0306 (8)	−0.0016 (7)	0.0058 (6)	−0.0001 (7)
C6	0.0233 (7)	0.0256 (9)	0.0295 (8)	−0.0013 (7)	0.0059 (6)	0.0014 (7)
O7	0.0262 (6)	0.0428 (8)	0.0325 (6)	−0.0009 (6)	0.0088 (5)	−0.0056 (6)
O8	0.0309 (6)	0.0268 (6)	0.0286 (6)	0.0060 (6)	0.0077 (5)	0.0009 (5)
C9	0.0291 (8)	0.0228 (8)	0.0276 (8)	0.0000 (7)	0.0050 (6)	−0.0006 (6)
C10	0.0226 (7)	0.0295 (9)	0.0286 (8)	−0.0018 (7)	0.0064 (6)	−0.0018 (7)
O11	0.0252 (5)	0.0416 (7)	0.0302 (6)	0.0068 (6)	0.0047 (4)	−0.0076 (6)
C12	0.0251 (7)	0.0240 (8)	0.0319 (8)	0.0009 (7)	0.0061 (6)	−0.0012 (7)
O13	0.0285 (6)	0.0316 (7)	0.0305 (6)	0.0082 (6)	0.0010 (5)	−0.0051 (5)
C14	0.0267 (7)	0.0231 (8)	0.0302 (8)	0.0019 (7)	0.0056 (6)	−0.0024 (7)
C15	0.0291 (8)	0.0301 (9)	0.0380 (9)	−0.0033 (8)	0.0095 (7)	−0.0071 (8)
C16	0.0298 (9)	0.0393 (11)	0.0432 (11)	−0.0038 (8)	0.0021 (8)	−0.0154 (9)
C17	0.0419 (10)	0.0367 (10)	0.0319 (8)	0.0061 (9)	0.0014 (7)	−0.0079 (9)
C18	0.0423 (10)	0.0343 (10)	0.0344 (9)	0.0016 (9)	0.0083 (8)	0.0017 (8)
C19	0.0314 (8)	0.0291 (9)	0.0363 (9)	−0.0040 (8)	0.0068 (7)	0.0011 (8)

### Geometric parameters (Å, °)

**Table:**

O1—C2	1.207 (2)	C10—O11	1.416 (2)
C2—O3	1.344 (2)	C10—H101	0.984
C2—C6	1.518 (2)	O11—C12	1.4148 (19)
O3—C4	1.4628 (19)	C12—O13	1.435 (2)
C4—C5	1.530 (2)	C12—C14	1.499 (2)
C4—C10	1.524 (2)	C12—H121	0.987
C4—H41	0.991	C14—C15	1.388 (2)
C5—C6	1.517 (2)	C14—C19	1.392 (3)
C5—O8	1.436 (2)	C15—C16	1.390 (3)
C5—H51	0.995	C15—H151	0.969
C6—O7	1.406 (2)	C16—C17	1.384 (3)
C6—H61	1.014	C16—H161	0.981
O7—H71	0.861	C17—C18	1.388 (3)
O8—C9	1.417 (2)	C17—H171	0.977
C9—C10	1.540 (2)	C18—C19	1.390 (3)
C9—O13	1.4088 (19)	C18—H181	0.963
C9—H91	1.005	C19—H191	0.983
			
O1—C2—O3	121.52 (15)	C9—C10—O11	104.69 (13)
O1—C2—C6	128.19 (15)	C4—C10—O11	111.49 (15)
O3—C2—C6	110.28 (13)	C9—C10—H101	113.3
C2—O3—C4	110.88 (12)	C4—C10—H101	111.0
O3—C4—C5	104.62 (13)	O11—C10—H101	111.9
O3—C4—C10	109.58 (14)	C10—O11—C12	107.49 (12)
C5—C4—C10	105.37 (13)	O11—C12—O13	104.83 (13)
O3—C4—H41	111.7	O11—C12—C14	110.64 (13)
C5—C4—H41	112.9	O13—C12—C14	111.54 (15)
C10—C4—H41	112.2	O11—C12—H121	110.1
C4—C5—C6	103.48 (12)	O13—C12—H121	108.5
C4—C5—O8	103.80 (13)	C14—C12—H121	111.0
C6—C5—O8	109.98 (14)	C12—O13—C9	108.59 (12)
C4—C5—H51	112.3	C12—C14—C15	119.63 (16)
C6—C5—H51	115.8	C12—C14—C19	120.33 (15)
O8—C5—H51	110.7	C15—C14—C19	120.04 (16)
C2—C6—C5	102.55 (12)	C14—C15—C16	120.10 (18)
C2—C6—O7	109.20 (14)	C14—C15—H151	120.4
C5—C6—O7	117.93 (14)	C16—C15—H151	119.5
C2—C6—H61	106.9	C15—C16—C17	119.99 (17)
C5—C6—H61	107.1	C15—C16—H161	120.7
O7—C6—H61	112.2	C17—C16—H161	119.3
C6—O7—H71	103.8	C16—C17—C18	119.95 (17)
C5—O8—C9	108.88 (13)	C16—C17—H171	120.4
O8—C9—C10	106.84 (14)	C18—C17—H171	119.6
O8—C9—O13	113.45 (14)	C17—C18—C19	120.40 (18)
C10—C9—O13	104.58 (13)	C17—C18—H181	118.6
O8—C9—H91	109.2	C19—C18—H181	121.0
C10—C9—H91	114.3	C14—C19—C18	119.51 (17)
O13—C9—H91	108.5	C14—C19—H191	118.2
C9—C10—C4	104.06 (13)	C18—C19—H191	122.3

### Hydrogen-bond geometry (Å, °)

**Table:**

*D*—H···*A*	*D*—H	H···*A*	*D*···*A*	*D*—H···*A*
C4—H41···O1^i^	0.99	2.37	3.200 (3)	141
C6—H61···O8^ii^	1.01	2.49	3.289 (3)	135
C9—H91···O1^iii^	1.01	2.55	3.349 (3)	137
C15—H151···O11^iv^	0.97	2.59	3.281 (3)	128
C16—H161···O13^v^	0.98	2.51	3.350 (3)	143
O7—H71···O1^iv^	0.86	1.97	2.811 (3)	165

Symmetry codes: (i) −*x*+2, *y*−1/2, −*z*+2; (ii) −*x*+1, *y*−1/2, −*z*+2; (iii) −*x*+2, *y*+1/2, −*z*+2; (iv) *x*−1, *y*, *z*; (v) −*x*, *y*−1/2, −*z*+1.
